# Emerging infectious diseases among indigenous peoples.

**DOI:** 10.3201/eid0707.017732

**Published:** 2001

**Authors:** J C Butler, S Crengle, J E Cheek, A J Leach, D Lennon, K L O'Brien, M Santosham

**Affiliations:** Centers for Disease Control and Prevention, Anchorage, Alaska 99516, USA. jbutler@cdc.gov


### Emerging Infectious Diseases among Indigenous Peoples

**Jay C. Butler,* Sue Crengle,† James E. Cheek,‡ Amanda J. Leach,§ Diana Lennon,¶ Katherine L. O'Brien,† and Mathuram Santosham†**

*Centers for Disease Control and Prevention, Anchorage, Alaska, USA; †Johns Hopkins University, Baltimore, Maryland, USA; ‡Indian Health Service, Albuquerque, New Mexico, USA; §Menzies School of Health Research, Casuarina, Northern Territory, Australia; and ¶Auckland University Medical School, Auckland, New Zealand

Many indigenous peoples are at higher risk for emerging infectious diseases compared to other populations. This conference panel focused on diseases of particular concern to Native Americans (American Indians and Alaska Natives), Australian aboriginal peoples, and the Maori of New Zealand. Important emerging diseases among these groups include respiratory tract infections, infections with antimicrobial-resistant organisms, zoonotic diseases, viral hepatitis, *Helicobacter pylori* and respiratory syncytial virus infections, diseases caused by Group A and B streptococcus, tuberculosis, and bacteremia and meningitis caused by *Streptococcus pneumoniae, Haemophilus influenzae* type b, and *Neisseria meningitidis*. Although the populations discussed are diverse, they have many things in common, including a high risk for many emerging infectious diseases, the requirement for culturally appropriate prevention and control strategies, and the need for increased leadership within communities of indigenous peoples.

### Native Americans

Native Americans comprise over 500 American Indian and Alaska Native tribes of unique ethnic and anthropologic origin. Although Native Americans account for only about 1% of the total U.S. population, American Indians account for a larger percentage of the population in western states, and Alaska Natives make up 17% of persons living in Alaska.

Hospitalization rates among American Indians are 20 to 40 times greater than rates in the general U.S. population for a number of zoonotic and vectorborne diseases, such as hantavirus pulmonary syndrome, plague, and Rocky Mountain spotted fever. Multiple factors may contribute to higher risks for American Indians, but a likely explanation is that they live in rural areas or do some type of agricultural work, both of which increase their chance of contact with small mammals and arthropods capable of transmitting these diseases. Greater understanding of the transmission of these diseases will help in the development of prevention strategies that also preserve traditional practices.

As with hantavirus pulmonary syndrome, methicillin-resistant *Staphylococcus aureus* (MRSA) infection acquired outside of healthcare settings is an emerging infectious disease first recognized among Native Americans. At some rural clinics serving Natives, over 60% of *S. aureus* isolates are methicillin-resistant. In one rural American Indian community, 74% of MRSA infections could not be linked to any of the known risk factors for MRSA such as hospitalization within the prior year, residence in a long-term care facility, hemodialysis, or injecting drug use.

Tuberculosis is an example of a disease that recently reemerged, is now in decline, but continues to disproportionately affect Native Americans, both in number of cases and severity of disease. Annual incidence for Native Americans remains twice that of the overall U.S. population, and mortality rates are six times higher. Possible reasons for the persistence of tuberculosis among Natives include living in crowded households, high rates of type 2 diabetes mellitus, and deterioration of the public health infrastructure.

The epidemiology of invasive pneumococcal disease has been characterized for three groups of Native Americans—Alaska Natives, members of the Navajo Nation, and White Mountain Apaches. Rates of invasive infection among Native American children <2 years old are some of the highest reported in the world. Across all age groups, rates are generally higher for Native Americans compared with those for white or black persons in the United States. The distributions of pneumococcal serotypes that cause invasive disease among Navajo and Alaska Native children differ from distributions among the total U.S. population. From 1989 to 1996, only 68% of sterile site isolates collected from Navajo children <2 years old were serotypes in the licensed 7-valent pneumococcal conjugate vaccine. Similarly, only 74% of isolates from Alaska Natives <2 years old were vaccine serotypes. This compares with 83% for non-Native children living in Alaska and 82% for the total U.S. population. Few studies have been conducted to determine the reason for increased disease rates or different serotype distribution for Native Americans. Among Alaska Native infants, group childcare, living with someone who chews tobacco, and lack of breastfeeding were factors associated with invasive pneumococcal disease. A study of risk factors among Navajo adults is underway.

It is not clear whether data from one Native American group can be applied to other tribal populations. Currently, no published data identify specific genetic factors among Native Americans that may contribute to an increased risk of contracting pneumococcal disease. Efforts to reduce the burden of pneumococcal disease among Native Americans include use of 23-valent polysaccharide vaccine in adults and 7-valent conjugate vaccine in infants, judicious antimicrobial

Address for correspondence: Jay C. Butler, 4055 Tudor Centre Drive, Anchorage, AK 99516, USA; fax: 404-638-5566; e-mail: jbutler@cdc.gov drug use to limit spread of drug-resistant strains, reducing the incidence of conditions and activities associated with greater risk of infection (diabetes mellitus, alcohol abuse, cigarette smoking), and support of programs to increase knowledge about and use of protection measures against acute respiratory infection such as breast-feeding.

### Australian Aboriginal Peoples

Aboriginal peoples account for 2% of the population of Australia. Aboriginal children have extremely high rates of chronic suppurative otitis media (CSOM) and purulent rhinitis. In a longitudinal study in one remote community, 60% of infants developed otorrhea by 6 months of age. As a result, substantial hearing loss occurs in at least 20% to 25% of school-age children and is associated with diminished school performance. Longitudinal studies of aboriginal infants have shown that otitis media often develops within weeks of birth, only days after initial colonization with the three principal bacterial pathogens *S. pneumoniae, H. influenzae,* and *Moraxella catarrhalis*. Multiple serotypes and molecular subtypes cocolonize infants and contribute to persistent and progressive middle ear disease. This multiplicity of colonizing organisms, and the high density of colonizing bacteria in the nasopharynx, may create a vicious cycle of inflammation in the upper airway and clinically manifest as CSOM and purulent rhinitis. The density and diversity of pathogens may also limit the success of antimicrobial therapy.

Colonization by bacterial respiratory pathogens among aboriginal children in the absence of selective pressure from antimicrobial agents is characterized by chronic infection from an early age. Bacterial competition, genetic exchange among cocolonizing organisms, and limited immune responses influence which colonizing strains dominate. Selective pressure from antibiotics provides a window of opportunity for resistant, "hidden" clones to become dominant and spread among members of the community. In a study utilizing nasopharyngeal swabs and microbiologic media, which permitted drug-resistant and susceptible pneumococcal colonies to be quantitated, densities were 2 to 3 log lower for resistant strains compared with susceptible strains in children not treated with antibiotics. However, during antibiotic treatment, the resistant strains became dominant and remained the dominant strains for several months, providing increased opportunity for transmission to susceptible persons.

In another aboriginal community, widespread use of single-dose azithromycin for trachoma was followed by a rapid increase in carriage of macrolide-resistant pneumococci. Compared with persons not colonized with pneumococci, those colonized before use of azithromycin were at greater risk of having macrolide-resistant strains isolated from the nasopharyngeal specimens after azithromycin use. Similar results may be expected from vaccine-induced immunologic selective pressures, although the clinical impact of this phenomenon will require careful evaluation.

### Maori of New Zealand

The Maori account for approximately 16% of the population of New Zealand. Although the health of non-Maori, non-Pacific Polynesian children is comparable to that of children in other western countries, poorer health statistics, particularly for infectious diseases, are noted for Maori children. Humanitarian government policies (Social Security Act of 1938, free public hospitals, subsidized housing) likely contributed to improvement in the Maori infant mortality rate, but some disparity continues, most likely because of fewer educational and economic opportunities. Epidemics of vaccine-preventable diseases continue because vaccination rates are lower for the Maori than for other residents of New Zealand. Hospitalizations for pneumonia and bronchiolitis, mainly caused by respiratory syncytial virus, overload the healthcare system each winter.

A continuing, 10-year clonal epidemic of *Neisseria meningitidis* serogroup B disease (B4:P1.4) involving mostly Maori and Pacific Island children (more recent immigrants to New Zealand) highlights the importance of overcrowded households as a factor in transmission of meningococcal infection. Provision of more suitable yet affordable housing may reduce transmission of meningococcal and other infectious diseases. Preliminary data look promising that a strain-specific vaccine may control the epidemic.

Rheumatic fever has continued to be a problem among the Maori, as well as Pacific Island children. Prevention of rheumatic fever is being addressed by study of an intensive program in schools where throat swabs are collected for culture and penicillin therapy is provided for children with *Streptococcus pyogenes*.

### Community Perspective and Future Directions

All too often, health research conducted among indigenous peoples has not incorporated their local worldview, cultural beliefs, or practices. As a result, research questions more often reflect researchers' interests and needs rather than those of the community. Study designs and data collection may have little meaning to community residents and therefore limit scientists' ability to obtain informed consent and collect accurate data. Researchers may have no sense of accountability for either the community's or the participant's data, and, after data collection is complete, the research team is never seen in the community again. Finally, research conducted and data collected have not always been used to bring about positive outcomes within the community and improve the health of indigenous peoples. Thus, community residents may feel that research is conducted *on* them rather than *with* them.

Participation by members of indigenous communities in research is increasing. However, to date, their contribution is mostly limited to providing community access, assisting with data collection, and facilitating ethical and institutional review board approvals. To perform research that will address the health issues of greatest concern among indigenous peoples, increasing community involvement is needed to select research topics, develop hypotheses and research questions, identify optimal study designs, create and implement data collection instruments, and analyze and interpret data. To this end, professional development of health researchers in indigenous communities is imperative.

